# Validation of the Korean Version of Culturally Responsive Experiences in Close Relationships–Short Form

**DOI:** 10.1007/s10447-023-09503-6

**Published:** 2023-02-18

**Authors:** Ji-yeon Lee, Yun-Kyung Kim, Yun-Jeong Shin

**Affiliations:** 1 Graduate School of Education, Hankuk University of Foreign Studies, Seoul, Republic of Korea; 2 Department of Education, Graduate School of Education & Information Studies, University of California, Los Angeles, CA USA; 3 Department of Education, Seoul National University, Kwanak-Ro 1, Kwanak-Gu, Seoul, Republic of Korea

**Keywords:** Attachment, ECR-R, Short form, Rasch analysis, Validation, Korean version

## Abstract

The authors developed and validated the Korean version of the Experiences in Close Relationships—Short Form (K-ECRR-SF) with the goal of developing a culturally responsive scale. In study 1, a Rasch analysis was conducted on the 36 original items in the ECR-Revised (ECR-R) to select items that best represent anxiety and avoidance subscales by considering cultural equivalence. In study 2, confirmatory factor analysis (CFA) was conducted for the selected 12 items with a different sample. The factor structures of the ECR-R and K-ECRR-SF through CFA were then compared through CFA. In addition, the K-ECRR-SF items were tested for related constructs (i.e., reassurance and support seeking, loneliness, dyadic satisfaction, depression, anxiety, and fear of intimacy) to its criterion evidence. The newly developed K-ECRR-SF is confirmed to be valid and culturally responsive scale in measuring attachment in Korea.

## Introduction

The Coronavirus 2019 pandemic has increased the physical distance among people, and in these difficult times, the quality of relationship has become more important. A barometer of the quality of relationship is attachment, which can be defined as an emotional bonding between individuals and their attachment figures (Bowlby, 1969). In addition, attachment influences how people think and behave in intimate relationships (Hazan & Shaver, 1987). Despite the consensus regarding its importance in different aspects of individuals, efforts for its measurements continue to evolve, especially in cross-cultural contexts.

Attachment style was originally measured as a categorical variable. Bartholomew and Horowitz (1991) developed four attachment categories (secure, fearful, preoccupied, and dismissive) that had been measured using Relationship Questionnaire (Hazan & Shaver, 1987) for a long time. With the limitations of using categorical variables in empirical studies, the Experiences in Close Relationships (ECR) scale (Brennan et al., 1998) assesses individual differences in attachment style using anxiety and avoidance as two continuous measures (18 items each). The ECR scale has shown strong psychometric properties across studies (e.g., Mikulincer & Shaver, 2007). Despite being a highly reliable and valid measure scores, this scale is quite long, which is not ideal for empirical research and broader clinical usage. This problem was addressed with the development of ECR-Short Form (ECR-S: Wei et al., 2007) that has 6 items each for anxiety and avoidance. Overall, ECR-S shows acceptable evidence of validity and reliability across studies carried out mostly in Western countries (Alonso-Arbiol et al., 2007; Wei et al., 2007).

## Cultural Equivalence

Cross-cultural studies on attachment take an etic approach, applying the attachment theory developed in Western society to non-Western cultures (Cassidy & Shaver, 2008). Attachment theory has an evolutionary nature (Bowlby, 1969), and thus regarded as a universal phenomenon. Although the function of attachment is universal, culture-specific contexts must be considered in understanding which types of attachment are adaptive in a given context (Main, 1990). Specifically, in Korea, Lee et al. (2008) found that items measuring high levels of anxiety and avoidance may not be appropriate for the sample of Korean college students because they are not experienced or difficult to report. Therefore, whether the underlying construct assessed by an instrument has the same meaning in each culture requires re-assessment of the construct. Reflecting culture-specific prototypes of attachment requires the selection of culturally appropriate items in shortening the ECR-Revised (ECR-R).

The most frequent method for achieving cultural equivalence when revising established English measures into other languages is a three-step procedure of translation, back-translation, and verification (Brislin et al., 1973). In most countries, such as Greece (Tsagarakis et al., 2007), Germany (Ehrenthal et al., 2009), Serbia (Hanak & Dimitrijevic, 2013), Romania (Rotaru & Rusu, 2013), Italy (Busonera et al., 2014), and Korea (Kim, 2004), ECR-R is merely translated and used after testing its validity and reliability.

There are some countries where the ECR-R was created in their own culture. The Thai ECR-R was translated from the original English version, and 18 items that best represent attachment anxiety and avoidance construct in Thailand were selected (Wongpakaran & Wongpakaran, 2012). To develop a shorter Czech version (ECR-R-16: Kaščáková et al., 2016), clinicians selected the best items among the 36 items of the ECR-R that show adequate psychometric properties. Germany (Brenk-Franz et al., 2018) and Spain (Fernández-Fuertes et al., 2011) also selected their own best items in shortening the ECR-R. The selected items represent the main components of attachment anxiety and avoidance. We compared the differences of the selected items for the short versions of ECR-R in different countries (see Appendix Table 4). Meanwhile, ECR-S has not been widely tested for cultural equivalence.

The Korean ECR-S was tested after its translation but the reliability of attachment anxiety and avoidance was not satisfactory (Lee & Shin, 2019). Another study was conducted in South Korea to develop the shortened version of ECR-R-K (Kim, 2004), ECR-R-K14 (Yun et al., 2017), to assess the attachment style of patients. However, the sample was collected among medical school students, and their DIF (Differential Item Functioning) analysis did not test for discriminatory items with respect to gender.

## Gender Differences

Gender difference in adult attachment must also be considered in selecting the best items in a given culture. Several studies (Van IJzendoorn & Bakermans-Kranenburg, 2010) found no gender difference in the attachment styles (dismissive or preoccupied) on the basis of meta-analysis of 65 samples across cultures (Cassidy & Shaver, 2008) while others found gender differences in attachment styles and relationship satisfaction on the basis of empirical findings of a meta-analysis with 113 samples from 100 studies (Giudice, 2011a, b). For example, males have a higher tendency to attachment avoidance with potential benefit of lowering commitment while female are more anxiously attached to promote commitment in relationships (Del Giudice, 2019). In terms of perceived quality of relationship, that of females tends to be more related to the degree of their partners’ comfort with closeness, but that of males tends to be more related to their partners’ anxiety about being unloved or abandoned (Collins & Read, 1990). In other words, emotional closeness is more important for females in general, whereas self-reliance is more important for males. This finding may reflect traditional gender norms.

Regarding the possible gender differences in romantic relationships, cultural norms on traditional gender roles must also be considered. For example, in South Korea, traditional Confucianism is revealed in the principle of Thrice Following—women should obey her father when she is young, her husband when she is married, and her son when she is old (Ko, 1994). This principle shows the perception that women are expected to be passive and committed to household work while seeking comfort and affection from men (Mann & Cheng, 2001). In modern South Korea, women have obtained higher education and are pursuing more prestigious jobs while seeking gender equity (Ahn, 2011). However, South Korean women are still implicitly affected by societal influences in gender norm, which may be manifested in their engagement in romantic relationships (Del Giudice, 2019). Therefore, we consider gender differences in selecting the best items for creating the Korean short version of ECR-R (K-ECRR-SF).

## Purpose of the Study

To represent anxiety and avoidance attachment among South Korean college students, we conducted a Rasch analysis to select the best items instead of merely translating the pre-selected ECR-S items developed within Western cultures. To evaluate the validity of the K-ECRR-SF, we performed two separate studies. Study 1 carried out Rasch analysis of the 36 original items in the ECR-R to select those that best capture the core component of attachment anxiety and avoidance of Koreans. Study 2 tested our hypothesis that ECR-R and K-ECRR-SF are comparable with respect to their construct and criterion evidence. To this end, we conducted a confirmatory factor analysis (CFA) for the selected items with a different sample, and tested if the newly selected ECR items correlated with existing relevant constructs (i.e., reassurance and support seeking, loneliness, dyadic satisfaction, depression, anxiety, and fear of intimacy). The correlations with relevant constructs were used as evidence of criterion validity of ECR-R, and likewise indicated criterion evidence of K-ECRR-SF. The factor structures of the ECR-R and K-ECRR-SF were then compared through CFA.

## Study 1

The purpose of study 1 was to select the most appropriate items to include in the K-ECRR-SF. From the Korean version of ECR-R (Kim, 2004), a combination of empirical and rational perspectives was adopted to determine which items to include in the K-ECRR-SF. Rasch analysis, a representative item response theory (IRT) modeling, was used to gather empirical evidence for item selection.

### Method

#### Procedure and Participants

The data were collected via an online survey company, Marketlink. Marketlink has over 500,000 online survey panels nationwide and panel membership are voluntarily in South Korea. After completing survey, panel members were given monetary incentives. The sample included undergraduate and graduate students that voluntarily registered to the nationwide panel and completed an online questionnaire. The survey specifically targeted those who identified themselves as college students to measure attachment to romantic partners in early adulthood, which marks the transition of attachment figure from parents to romantic partners (Mikulincer & Shaver, 2007). The sample included 165 participants, 77 male (47%) men and 88 female (53%) women, with age range 18–26 years (M = 21.24, SD = 2.19). This study was approved by the Institutional Review Board of the Hankuk University of Foreign Studies and complied with the Declaration of Helsinki. Informed consent was obtained from the participants prior to the survey.

#### Instrument

Adult attachment style was measured with the ECR-R (Kim, 2004) that adapted and validated the original ECR-R (Fraley et al., 2000) to Korean. Considering that this study intended to measure attachment to romantic partners, we partially modified the Korean version of the ECR-R (Kim, 2004). Kim (2004) referred to the attachment figure as “others”, which we redirected specifically to “a romantic partner”. The ECR-R is a 36-item self-report measure, with 18 items each for anxiety and avoidance subscales. In a 7-point scale ranging from 1 (strongly disagree) to 7 (strongly agree), participants responded the degree to which each statement describes their general orientation in romantic relationships.

#### Analysis Strategy

Rasch analysis is a psychometric technique that was developed and has been widely used to construct and validate instruments. Based on Thurstonian ideas on psychological item response, this analysis provides parameter estimates for persons’ ability (e.g., *anxiety or avoidance of each participant*) and item difficulty (e.g., *severity of anxiety or avoidance conveyed by each item*) that are not interdependent, thus allowing for a stable performance across settings and populations (Lord & Novick, 1968). Among the Rasch models that can be applied to polytomous items, partial credit model (PCM; Masters, 1982) was deemed the most appropriate for this study. PCM assumes that each item has its own response structure and thus freely estimates item-specific difficulty parameters, which is desirable in this study where there is little evidence of all items sharing the same response structure. The analysis was conducted using WINSTEPS software program (Linacre, 2020).

Based on the empirical evidence from Rasch analysis and the reasoning in line with the theoretical framework of ECR-R, the following four criteria were adopted to select the most appropriate items: 1) *Item fit*. Items with mean squares of fit statistics of 0.5–1.5 were considered to have acceptable fit as per previous criteria (Linacre, 2002); 2) *Rating scale validity*. Items with *ordered* and *fit* rating categories were considered to have functional response structure. These orders and fits of the rating categories were examined to check if the rating scales were ordinal and thus stayed true to the assumption that endorsing a *higher* rating category indicated a *higher* level of the underlying construct (Cordier et al., 2019); 3) *Gender-related differential item functioning (DIF)*. Items with significantly different item difficulty measures between males and females were reconsidered; 4) *Item difficulty and content*. We prioritized items that enable K-ECRR-SF to cover the largest possible continuum of anxiety or avoidance. A reasonable balance of content areas within each subscale was ensured to secure content coverage (Edelen & Reeve, 2007).

### Results and Discussion

#### Criterion 1. Item Fit

Items were examined for their fit to the PCM; *infit* and *outfit* statistics ranging 0.5–1.5 were considered to have acceptable fit, per criteria from Linacre (2020). *Infit* was sensitive to unexpected responses in items with a severity level *near to* a person’s anxiety or avoidance level, whereas *outfit* is sensitive to unexpected responses to items with a severity level *far from* a person’s anxiety or avoidance level. Hence, items 5, 9R, and 17 (infit = 1.6, 1.9, 1.6; outfit = 1.7, 2.2, 1.6) in anxiety subscale and items 19, 26R, and 27R (infit = 1.5, 1.4, 1.5; outfit = 1.8, 1.7, 1.7) in avoidance subscale were determined as underfit and were excluded in the short form.

#### Criterion 2. Rating Scale Validity

Items were examined for their rating scale validity via observed average and mean squares of outfit in each of the seven categories (See Table 1). Those with disordered and underfit categories were items 9R, 12, 13, 17, and 18 of anxiety subscale and items 19, 26R, 27R, 34R, 35R, and 36R of avoidance subscale.

**Table 1:** Rating scale validity of Anxiety and Avoidance subscale original form (18 items each)

Anxiety						Avoidance					
Item No.	Cat.	Freq.	Obs Avg	Obs Avg Diff.	Outfit MnSq	Item No.	Cat.	Freq.	Obs Avg	Obs Avg Diff.	Outfit MnSq
*Items with disordered and/or unfit categories*						*Items with disordered and/or unfit categories*					
9R	1	**5**	−1.11	–	1.4	19	1	28	−1.83	–	2.3
	2	8	−0.74	0.4	2.1		2	31	−1.13	0.7	1.0
	3	16	−0.21	0.5	3.2		3	21	−0.57	0.6	4.0
	4	36	−0.43	−0.2	2.1		4	19	−0.23	0.3	1.2
	5	40	−0.38	0.1	1.0		5	34	−0.16	0.1	1.9
	6	44	−0.36	0.0	2.1		6	21	0.26	0.4	1.4
	7	16	−0.63	−0.3	2.0		7	11	0.56	0.3	1.0
12	1	41	−0.92	–	0.9	26R	1	11	−3.23	–	1.1
	2	62	−0.52	0.4	0.9		2	42	−0.89	2.3	1.3
	3	27	−0.17	0.4	0.5		3	44	−0.53	0.4	1.0
	4	27	−0.02	0.2	1.2		4	46	−0.04	0.5	1.4
	5	4	0.11	0.1	1.1		5	16	0.14	0.2	1.2
	6	3	1.09	1.0	0.5		6	3	0.76	0.6	0.9
	7	1	0.12	−1.0	2.4		7	3	−0.94	−1.7	5.8
13	1	38	−0.93	–	1.0	27R	1	11	−3.21	–	1.1
	2	46	−0.45	0.5	1.5		2	50	−0.83	2.4	1.3
	3	32	−0.33	0.1	0.6		3	41	−0.53	0.3	1.1
	4	24	−0.20	0.1	1.7		4	41	0.04	0.6	1.4
	5	19	0.09	0.3	1.4		5	18	0.21	0.2	1.2
	6	5	−0.28	−0.4	5.3		6	2	−0.48	−0.7	2.9
	7	1	−0.59	−0.3	3.7		7	2	−1.06	−0.6	7.1
17	1	15	−1.00	–	1.8	34R	1	8	−4.14	–	0.5
	2	20	−0.72	0.3	1.8		2	25	−1.44	2.7	1.0
	3	12	−0.37	0.4	2.2		3	40	−0.68	0.8	0.8
	4	22	−0.56	−0.2	1.2		4	62	−0.12	0.6	0.8
	5	57	−0.39	0.2	2.5		5	21	0.37	0.5	0.8
	6	27	−0.08	0.3	1.2		6	8	0.31	−0.1	1.2
	7	12	−0.08	0.0	1.2		7	1	−2.58	−2.9	6.8
18	1	65	−0.72	–	1.2	35R	1	25	−1.87	–	1.4
	2	53	−0.45	0.3	0.6		2	62	−0.69	1.2	1.3
	3	29	−0.07	0.4	0.6		3	41	−0.27	0.4	1.0
	4	9	0.18	0.3	0.7		4	24	0.12	0.4	1.2
	5	6	0.08	−0.1	2.3		5	11	0.16	0.0	1.7
	6	3	−0.29	−0.4	3.8		6	1	−0.88	−1.0	5.1
	7	–	–	–	–		7	1	0.28	1.2	1.9
*Items with ordered and fit categories*						36R	1	10	−3.36	−	1.3
1	1	14	−1.28	–	0.9		2	31	−1.20	2.2	1.1
	2	27	−0.84	0.4	1.3		3	39	−0.64	0.6	1.1
	3	25	−0.79	0.0	0.4		4	65	−0.01	0.6	0.7
	4	27	−0.37	0.4	0.3		5	12	−0.14	−0.1	2.5
	5	49	−0.16	0.2	0.9		6	6	−0.02	0.1	1.8
	6	20	0.27	0.4	0.7		7	2	0.92	0.9	0.8
	7	3	0.36	0.1	1.1	*Items with ordered and fit categories*					
2	1	21	−1.29	–	0.7	20R	1	14	−3.03	–	0.9
	2	44	−0.73	0.6	1.1		2	47	−1.16	1.9	0.8
	3	19	−0.71	0.0	0.5		3	40	−0.35	0.8	0.6
	4	25	−0.27	0.4	0.3		4	35	0.00	0.4	1.3
	5	39	0.04	0.3	0.4		5	15	0.37	0.4	0.5
	6	16	0.37	0.3	0.7		6	9	0.54	0.2	1.0
	7	1	0.27	−0.1	1.0		7	5	0.80	0.3	0.8
3	1	27	−1.27	–	0.6	21	1	31	−1.91	–	1.3
	2	38	−0.82	0.5	0.4		2	50	−0.97	0.9	0.8
	3	21	−0.44	0.4	0.3		3	28	−0.26	0.7	0.6
	4	32	−0.17	0.3	0.3		4	26	0.26	0.5	0.3
	5	28	0.03	0.2	0.5		5	25	0.31	0.1	1.3
	6	16	0.42	0.4	0.6		6	5	0.85	0.5	0.8
	7	3	0.38	0.0	1.0		7	–	–	–	–
4	1	23	−1.35	–	0.6	22R	1	22	−2.34	–	1.1
	2	36	−0.85	0.5	0.4		2	56	−1.00	1.3	0.6
	3	22	−0.52	0.3	0.4		3	43	−0.15	0.8	0.5
	4	28	−0.25	0.3	0.3		4	31	0.28	0.4	0.5
	5	35	0.04	0.3	0.4		5	12	0.64	0.4	0.7
	6	18	0.22	0.2	0.8		6	1	1.37	0.7	0.6
	7	3	1.05	0.8	0.7		7	–	–	–	–
5	1	7	−1.47	–	0.9	23	1	21	−2.68	–	0.8
	2	8	−0.87	0.6	1.5		2	46	−0.92	1.8	1.1
	3	5	−0.49	0.4	1.8		3	31	−0.45	0.5	0.5
	4	35	−0.63	−0.1	1.1		4	22	0.07	0.5	0.8
	5	39	−0.36	0.3	1.4		5	33	0.26	0.2	0.7
	6	51	−0.27	0.1	1.4		6	10	0.42	0.2	1.8
	7	20	−0.09	0.2	1.3		7	2	1.05	0.6	0.8
6	1	21	−1.30	–	0.7	24	1	28	−2.16	–	0.9
	2	27	−0.64	0.7	1.2		2	42	−1.00	1.2	0.7
	3	36	−0.46	0.2	0.5		3	22	−0.52	0.5	1.7
	4	41	−0.24	0.2	0.9		4	33	0.07	0.6	0.5
	5	24	−0.21	0.0	1.5		5	27	0.18	0.1	1.2
	6	16	0.28	0.5	1.7		6	10	0.78	0.6	0.7
	7	–	–	–	–		7	3	0.78	0.0	0.9
7	1	22	−1.21	–	0.8	25	1	41	−1.74	–	1.0
	2	39	−0.68	0.5	0.9		2	49	−0.77	1.0	1.1
	3	22	−0.47	0.2	0.6		3	22	−0.31	0.5	0.6
	4	37	−0.33	0.1	1.2		4	25	0.25	0.6	0.4
	5	27	−0.07	0.3	0.9		5	21	0.32	0.1	0.9
	6	13	0.21	0.3	1.1		6	5	1.04	0.7	0.4
	7	5	0.65	0.4	0.9		7	2	0.67	−0.4	1.1
8	1	17	−1.48	–	0.5	28R	1	10	−3.78	–	0.6
	2	32	−0.87	0.6	0.6		2	37	−1.46	2.3	0.6
	3	21	−0.54	0.3	0.8		3	48	−0.35	1.1	0.8
	4	41	−0.28	0.3	0.4		4	34	−0.10	0.2	0.7
	5	33	−0.05	0.2	0.5		5	18	0.38	0.5	0.5
	6	18	0.27	0.3	1.0		6	14	0.39	0.0	1.5
	7	3	0.35	0.1	1.0		7	4	0.88	0.5	0.8
10	1	42	−1.00	–	0.9	29R	1	14	−3.32	–	0.6
	2	45	−0.57	0.4	0.6		2	46	−1.08	2.2	0.8
	3	24	−0.22	0.4	0.4		3	63	−0.22	0.9	0.5
	4	29	−0.11	0.1	1.0		4	31	0.20	0.4	0.8
	5	16	−0.04	0.1	1.2		5	10	0.69	0.5	0.8
	6	6	0.55	0.6	0.5		6	1	0.28	−0.4	1.4
	7	3	0.49	−0.1	1.9		7	–	–	–	–
11R	1	19	−1.22	–	0.8	30R	1	4	−4.84	–	1.2
	2	44	−0.74	0.5	0.9		2	21	−1.95	2.9	1.2
	3	38	−0.49	0.3	0.5		3	28	−1.01	0.9	1.2
	4	34	−0.03	0.5	0.3		4	25	−0.35	0.7	1.3
	5	22	−0.02	0.0	1.0		5	32	−0.12	0.2	0.6
	6	6	0.46	0.5	1.4		6	37	−0.08	0.0	2.0
	7	2	0.82	0.4	0.8		7	18	0.43	0.5	1.2
14	1	49	−0.84	–	1.0	31R	1	9	−4.21	–	0.5
	2	61	−0.48	0.4	0.6		2	41	−1.41	2.8	0.5
	3	23	−0.21	0.3	1.1		3	45	−0.32	1.1	0.7
	4	16	−0.06	0.2	1.1		4	43	0.04	0.4	0.5
	5	9	0.08	0.1	0.9		5	18	0.40	0.4	0.9
	6	6	0.88	0.8	0.6		6	9	0.53	0.1	1.0
	7	–	–	–	–		7	–	–	–	–
15	1	29	−1.02	–	1.2	32	1	30	−1.85	–	1.1
	2	30	−0.75	0.3	0.9		2	55	−0.97	0.9	1.0
	3	18	−0.62	0.1	0.6		3	26	−0.26	0.7	0.7
	4	27	−0.22	0.4	0.6		4	24	0.27	0.5	0.7
	5	36	−0.13	0.1	1.5		5	22	0.30	0.0	0.8
	6	19	−0.04	0.1	1.4		6	7	0.76	0.5	0.8
	7	6	0.48	0.5	1.2		7	1	0.92	0.2	0.9
16	1	20	−1.11	–	1.1	33R	1	10	−3.80	–	0.6
	2	33	−0.74	0.4	1.3		2	44	−1.34	2.5	0.6
	3	33	−0.39	0.4	0.9		3	46	−0.32	1.0	0.6
	4	28	−0.27	0.1	0.7		4	50	0.06	0.4	0.6
	5	36	−0.19	0.1	1.1		5	10	0.69	0.6	0.6
	6	11	−0.07	0.1	1.8		6	5	1.05	0.4	0.6
	7	4	0.74	0.8	0.9		7	–	–	–	–

Cat. = Category (Category 1 = strongly disagree, 2 = disagree, 3 = slightly disagree, 4 = neutral, 5 = slightly agree, 6 = agree, 7 = strongly agree); Freq. = Frequency; Obs Avg = Observed average of person measure; Obs Avg Diff. = Observed average difference from the preceding category (For a rating scale to be ordered, the observed average difference should all be positive.); Outfit MnSq = mean square of outfit (For a rating scale to be fit, the mean squares of response categories should range between 0.5 and 1.5.)

#### Criterion 3. Gender-related DIF

Items with statistically significant gender-related DIF included item 13 (Mantel chi-square = 7.44, *p* < 0.05) and item 16 (Mantel chi-square = 19.15, *p* < 0.001) in the anxiety subscale and item 30R (Mantel chi-square = 7.59,* p* < 0.05) in the avoidance subscale, Based on criteria 1, 2, and 3, eight items from anxiety subscale (i.e., 5, 9R, 12, 13, 16, 17, and 18) and six items from avoidance subscale (i.e., 19, 26R, 27R, 30R, 34R, 35R, and 36R) were removed.

#### Criterion 4. Item Difficulty and Item Contents

**Fig. 1:**
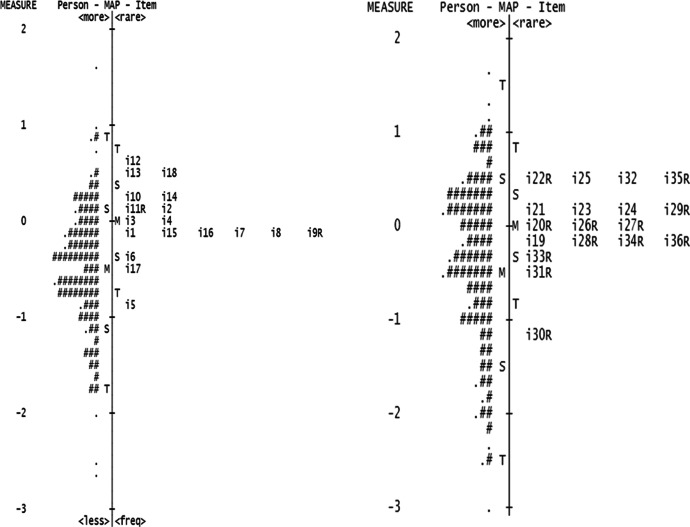
Wright Map of Anxiety and Avoidance Subscales-Full Form. Note. The left column and right columns correspond to Anxiety and Avoidance subscales, respectively. In each column, #’s represent persons’ severity levels and item numbers (e.g., i12) represent item difficulty levels

Among the remaining 10 items in the anxiety subscale, to maximize the continuum of anxiety measurable by the short form (see left-hand column of Fig. 1), we prioritized and retained one item each from 10 and 14, 2 and 11R, 3 and 4, and 6 for each difficulty range. Subsequently, for the selection, item contents were considered. The items of anxiety subscale were largely in three domains: (a) fear of rejection by one’s partner; (b) excessive need for approval from one’s partner; and (c) distress from one’s partner’s unresponsiveness. Each item represented one or more of the three domains, and we retained at least two items in each domain for the short form. Finally, based on the quantitative analysis of item difficulty and the qualitative analysis of item contents, the following conclusions were drawn. Item selection in domain (a) included 11R and 14. In domain (b), 4 and 10 were prioritized to retain the range of measurement. In domain (c), 7 and 8 that represent both *fear of rejection* and *frustration about unavailability* were chosen.

Among the remaining 12 items in the avoidance subscale, to maximize the continuum of anxiety measurable by the short form (see right-hand column of Fig. 1), we prioritized items 31R, 33R, 28R, and 20R. Subsequently, in terms of item contents, selected items fell largely under three domains: (a) fear of intimacy, (b) reluctance to dependency, and (c) reluctance to self-disclosure (Wei et al, 2007). Item selection included 22R and 24 in domain (a) and 29R and 33R in domain (b). In domain (c), items 20R and 28R that present the concept of avoiding self-disclosure with more concrete expression were chosen. As a result of Study 1, K-ECRR-SF is finalized to contain 12 items, 6 items each for anxiety and avoidance subscales (i.e., items 4, 7, 8, 10, 11R, and 14 for anxiety subscale, and items 20R, 22R, 24, 28R, 29R, and 33R for avoidance subscale).

## Study 2

Study 2 evaluated the validity and reliability of the K-ECRR-SF compared with those of the original form on the basis of classical test theory.

### Method

#### Participants and Procedure

The data were collected via an online survey company with compensated participants as described in study 1, the total of which was 150 for this study. Seven forms with majority of the items insincerely answered or left missing were omitted from analysis, yielding a final sample of 143 participants, 56 male (39%) male and 87 female (61%) female, with age range 18–28 years (M = 20.86, SD = 2.02).

#### Instrument

*Adult attachment style*. The ECR-R translated by Kim (2004) was modified in this study to narrow the attachment figure to romantic partners (36 items). Its short form, K-ECRR-SF, finalized in Study 1 included 12 items with 6 items reverse keyed. The internal consistency coefficient (Cronbach’s alpha) in this study 2 sample was 0.81 for the short form (Anxiety: 0.82, Avoidance: 0.86) and 0.92 for the original form (Anxiety: 0.92, Avoidance: 0.93).

*Reassurance Seeking*. Excessive Reassurance Seeking Scale (Joiner & Met al.,sky, 2001) was used, with four items rated in a 7-point-scale ranging from 1 (strongly disagree) to 7 (strongly agree). In this study 2 sample, the item scores were summed and their internal consistency coefficient was 0.88. A sample item would be ‘Do you find yourself often asking the people you feel close to how they *truly* feel about you?’.

*Support Seeking.* Support seeking subscale of the Berlin Social Support Scale (Schulz & Schwarzer, 2003) was used. It consists of five items in a 5-point-scale ranging from 1 (strongly disagree) to 5 (strongly agree). In this study 2 sample, the item scores were summed into a total score, and their internal consistency coefficient was 0.83.

*Loneliness*. The Revised UCLA Loneliness Scale (RULS: Russell et al., 1980) was used, with 20 items in a 4-point-scale from 1 (not at all) to 4 (often), among which 10 are reverse keyed. A sample item is ‘I feel left out’. The Korean version of RULS was validated by Kim and Kim (Kim & Kim, 1989) and showed acceptable reliability (internal consistency coefficient = 0.86). In this study 2 sample, the item scores were summed and their internal consistency was 0.89.

*Relationship Satisfaction*. Dyadic satisfaction subscale of Dyadic Adjustment Scale (DAS: Spanier, 1976) was used, with 10 items. Specifically, ratings were made on 7 items in a 6-point-scale from 0 (always) to 5 (never), 1 item in a 4-point-scale from 0 (never) to 4 (everyday), 1 item in a 7-point-scale from 0 (extremely unhappy) to 6 (perfect), and 1 item on relationship prospects in a 6-point-scale ranging from 0 (extremely pessimistic and unwilling to put effort) to 5 (extremely optimistic and willing to do anything). A sample item is ‘How often do you and your partner quarrel?’ The Korean version of DAS was validated and showed moderate reliability, internal consistency coefficient was 0.87 (Lee & Kim, 1996). In this study 2 sample, we summed the item scores and their internal consistency coefficient was 0.74.

*Depression*. Brief Patient Health Questionnaire-9 (PHQ-9: Kroenke et al., 2001) was used as a measure of depression severity with nine items rated in a 3-point-scale from 0 (not at all) to 3 (almost every day). A sample item is ‘Over the last 2 weeks, how often have you been bothered by the problem of feeling down, depressed, or hopeless?’ The Korean version of PHQ-9 was validated by An et al. (An et al., 2013) and showed acceptable reliability (internal consistency coefficient = 0.95). In this study 2 sample, we summed the item scores, and their internal consistency coefficient was 0.80.

*Anxiety*. In this study, the Beck Anxiety Inventory (BAI: Beck & Steer, 1990) Korean version (Kwon, 1992) was used, with 21 items rated in a 4-point-scale ranging from 0 (not at all) to 3 (severely). The items reflect symptoms of anxiety such as numbness or tingling, feeling hot, wobbliness in legs, etc. The internal consistency coefficient in study 2 was 0.89.

*Fear of intimacy*. The Fear of Intimacy Scale (FIS: Descutner & Thelen, 1991) is intended to assess individuals of their fear of intimacy whether they are currently in a relationship. A sample item is ‘I would feel uncomfortable telling the person (who would be in the close relationship with you) about things in the past that I have felt ashamed of.’ A total of 35 items (14 items reverse keyed) are rated in a 5-point-scale from 1 (not at all) to 5 (extremely). In study 2 sample, the internal consistency coefficient was 0.91.

#### Analysis Strategy

The reliability and validity of the short form, K-ECRR-SF, were assessed compared with the original form using Classical Test Theory approach. Evidence of internal structure was evaluated by using CFA. Fit indices of comparative fit index (CFI), Tucker-Lewis Index (TLI), root mean square error of approximation (RMSEA), standardized root mean square residual (SRMR) were used to assess model fit. Cutoff values close to 0.95 for CFI and TLI, 0.08 for SRMR, and 0.06 for RMSEA were required to conclude an adequate fit between the hypothesized model and the observed data (Hu & Bentler, 1999). Using Mplus 7 software program, we estimated the parameters by maximum likelihood method as the observed variables were confirmed of their normality (Kline, 2015). In addition, *convergent* validity was tested by inspecting the correlation with measures that are supposed to be *similar* with anxiety or avoidance attachment styles, and *discriminant* evidence was tested by inspecting the correlation with measures that are supposed to be *different* from anxiety or avoidance attachment styles.

### Results and Discussion

#### Correlation between Original and Short Form

Correlations between the original form (36 items) and short form (12 items) was high overall (*r* = 0.951, *p* < 0.001) and on a subscale level (Anxiety:* r* = 0.939, *p* < 0.001, Avoidance: *r* = 0.958, *p* < 0.001). Furthermore, the correlation between anxiety and avoidance subscales was* r* = 0.273 (*p* < 0.001) in the original form but decreased to *r* = 0.169 (*p* < 0.05) in the short form. This consequence was expected as only items that most evidently depict anxiety or avoidance were retained in each subscale. In other words, the items that simultaneously represent both anxiety and avoidance to a comparable degree were deprioritized to ensure that each subscale strongly characterized its own construct.

#### CFA

**Fig. 2:**
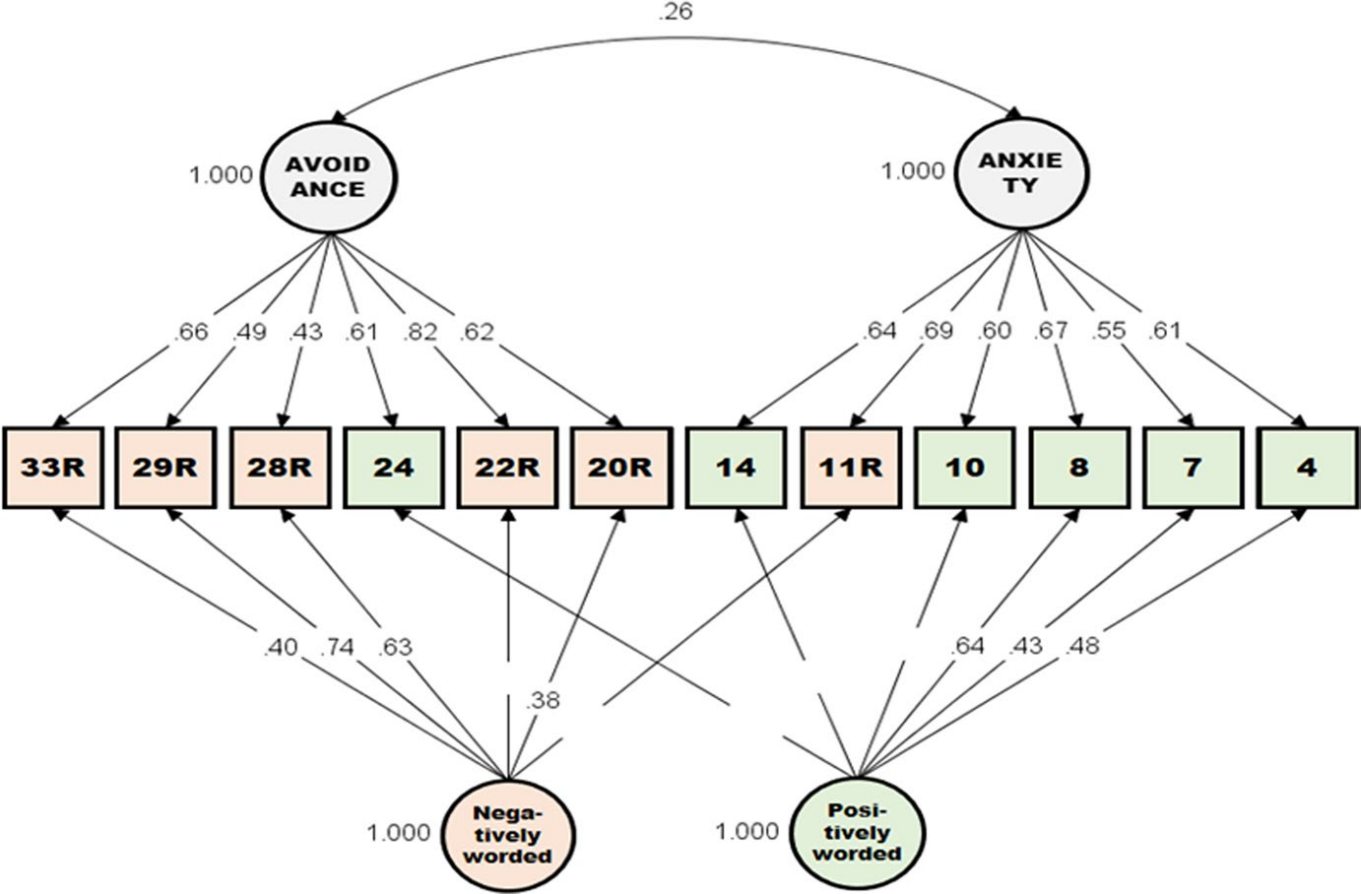
CFA Model of the Short Form with Latent Method Factors. Note. Statistically significant standardized factor loadings are displayed

A traditional two-factor (Anxiety and Avoidance) CFA model was used to compare the construct validity of the original form and that of the short forms (see Model 1 of Table 2). Numerous preceding studies on ECR or ECR-R found systematic errors caused by item wording style and correspondingly added method factors (e.g., positive and negative statements) to the model (Lee & Shin, 2019; Wei et al., 2007; Wongpakaran & Wongpakaran, 2012). That is, it was hypothesized that respondents have systematic orientations to answering positively and negatively worded items. Therefore, we added two *orthogonal* latent factors—positively worded items loaded on one factor and negatively worded (reverse keyed) items loaded on another factor (see Model 2 of Table 2). The results indicated that the short form demonstrated better model fit than the original form, with respect to both Models 1 and 2. In particular, the short form in Model 2 showed the best model fit, with indices that met all the recommended cutoffs (Hu & Bentler, 1999). All factor loadings of anxiety or avoidance and items were statistically significant, and those of method factors and not all items were statistically significant (See Fig. 2).

**Table 2:** Comparison of CFA

Scale	Model	X^2^	df	CFI	TLI	RMSEA (90% confidence interval)	SRMR
Short form	Model 1	119.78	53	0.902	0.878	0.094 (0.072–0.116)	0.084
	Model 2	58.03	41	0.975	0.960	0.054 (0.012–0.084)	0.056
Original form	Model 1	1982.79	593	0.612	0.588	0.128 (0.122–0.134)	0.146
	Model 2	1390.53	557	0.767	0.737	0.102 (0.096–0.109)	0.136

#### Convergent and Discriminant Evidences

Reassurance-seeking behavior was measured with positively and negatively worded items. The *positive* items were negatively correlated with avoidance subscale of both the short and original forms, indicating discriminant evidence in those with avoidance attachment style that do *not* find themselves seeking reassurance (see Table 3). The *negative* items were positively, albeit marginally, correlated with the overall and anxiety scores of the short and original forms, with the former having a slightly higher coefficient. Those who are anxious in their relationship are *likely* to be concerned about how others perceive them, regardless of how much they demonstrate reassurance-seeking behaviors. Similarly, support seeking was positively correlated with anxiety subscale and negatively correlated with avoidance subscale in both the short and original forms, which simultaneously indicated the convergent evidence of anxiety subscale and the discriminant evidence of avoidance subscale.

**Table 3:** Correlation of the short and original forms with related variables

	Short form			Original form		
	Overall	Anxiety	Avoidance	Overall	Anxiety	Avoidance
Reassurance seeking	0.03	0.15	−0.12	0.00	0.12	−0.13
positive	−0.10	0.07	−0.24^**^	−0.12	0.04	−0.25^**^
negative	0.21^*^	0.22^**^	0.09	0.18^*^	0.19^*^	0.09
Support seeking	−0.06	0.21^*^	−0.32^***^	−0.05	0.22^*^	−0.32^***^
Loneliness	0.45^***^	0.25^**^	0.45^***^	0.49^***^	0.28^***^	0.52^***^
Dyadic satisfaction	−0.54^***^	−0.33^***^	−0.52^***^	−0.55^***^	−0.36^***^	−0.52^***^
Depression	0.19^*^	0.19^*^	0.09	0.27^**^	0.25^**^	0.17^*^
Anxiety	0.27^**^	0.38^***^	0.01	0.31^***^	0.41^***^	0.06
Fear of intimacy	0.59^***^	0.19^*^	0.74^***^	0.63^***^	0.23^**^	0.80^***^

^***^p < 0.001, ^**^p < 0.01, ^*^p < 0.05

Moreover, the short and original forms and their subscales were positively correlated with loneliness and fear of intimacy but negatively correlated with dyadic satisfaction, with the correlation in avoidance subscales larger than that in anxiety subscales. By contrast, depression and anxiety were positively correlated with anxiety subscale of the short and original forms but not with the avoidance subscale of the short form. These results underpinned the key features of anxiety and avoidance. Avoidance more directly causes *social/interpersonal problems*, whereas anxiety is closely related to a maladaptive rumination that causes *psychological* distress (Lee & Seo, 2014). The results suggested that anxiety and avoidance subscales effectively measured their constructs and functioned well in both the short and original forms.

## Discussion

This research consists of two separate studies to develop the K-ECRR-SF. Developing this short Korean version of the ECR-R-SF questionnaire is critical in quantitative research to minimize participants’ burden of response while still capturing the major properties of the construct. To develop K-ECRR-SF, we adopted IRT(Item Responsive Theory) methods, a helpful methodology that independently estimates the parameters of person’s ability and item’s difficulty independently to provide abundant information on each item with less influence from population characteristics. Especially, Rasch models, a 1-parameter-logistic, is useful in that the parameters can be estimated stably with a relatively small sample and allows for direct interpretation. Therefore, using Rasch and content analyses in study 1, we selected six items each for anxiety and avoidance attachment under four criteria, namely, (1) item fit, (2) rating scale validity, (3) gender related DIF, and (4) item difficulty and item contents. We selected the items to equally represent all three attributes of anxiety attachment, which are (a) fear of rejection (11R and 14), (b) excessive need for appraisal from the partner (4 and 10), and (c) frustration from one’s partner’s unavailability (7 and 8). The items for avoidance attachment were also chosen by considering three major characteristics of avoidance attachment, (a) fear of intimacy (22R and 24), (b) excessive need for self-reliance (29R and 33R), and (c) reluctance to self-disclosure (20R and 28R).

During item selection and via DIF analysis, findings indicated gender differences in responses to attachment anxiety and avoidance items. The DIF analysis compares the responses of males and females with the same degree of severity level, thereby detecting items that demonstrate higher or lower difficulties for either of the gender groups with the same anxiety or avoidance levels. For attachment anxiety, males tend to respond with higher scores in perceiving that their partners changed their feelings with no apparent reason than females (item 13). Females were more likely to respond with higher scores in being upset for not obtaining the affection and support needed from one’s partner (item 16). For attachment avoidance, females showed higher difficulty in telling everything to their partners (item 30). Thus, we deleted those items with gender differences to remove the possible reflection of the shared gender stereotypes (Shields, 2002), such as women are more emotional and unpredictable, while men use the dismissed coping mechanism (Schmitt et al., 2003).

In study 2, the findings suggest that all 12 items in K-ECRR-SF have comparable psychological properties to the 36 items in the K-ECR-R. Specifically, the internal consistency of the 12 items of K-ECRR-SF is adequate for anxiety attachment (0.82), avoidance attachment (0.86), and overall ratings (0.81). These results demonstrate that the reliability of K-ECRR-SF is better than other previously developed ECR-S Korean versions (Lee & Shin, 2019; Yun et al., 2017). Specifically, this scale shows higher reliability (old K-ECR (Lee & Shin, 2019): anxiety- 0.81, avoidance -0.70; K-ECRR-SF (this study): anxiety—0.82, avoidance—0.86). In addition, the high correlation between the original and short forms imply that the selected items in K-ECRR-SF well portray both anxiety and avoidance regardless of item reduction.

The CFA results also show that a model with two oblique factors (anxiety and avoidance) along with two orthogonal factors (negative and positive phrasing) provides an adequate fit to the data for K-ECRR-SF. The factor loadings on anxiety and avoidance attachment were all above 0.4. Finally, sufficient evidence for convergent and discriminant validities were obtained by checking the correlation of anxiety and avoidance subscales with reassurance seeking, support seeking, depression, anxiety, loneliness, relationship satisfaction, and fear of intimacy. Similar patterns are observed on the correlations with K-ECRR-SF and those with ECRR. The results are consistent with several previous findings (Brenk-Franz et al., 2018; Fernández-Fuertes et al., 2011; Wei et al., 2007).

### Theoretical Implications

In testing the cultural equivalence of measurements, items of the original scale were mainly translated in previous literature and then used to test for equivalence. However, whether the underlying construct assessed by the instrument has the same meaning across cultures must be re-assessed. This study captured culture-specific prototypes of attachment in shortening the ECR-R in the Korean cultural context (K-ECRR-SF), instead of merely translating the ECR-S, which makes this measure superior to the one that translated and validated the original ECR-R (Lee & Shin, 2019).

Although the function of attachment is universal across cultures, cultural expressions of attachment anxiety and avoidance can differ culture (Lee et al., 2008). By developing culturally responsive attachment measurements, this study enables further studies in capturing culture-specific responses of attachment and relationship issues in Korea. Having the culturally responsive attachment measurement would enable to capture culture specific attachment related issues more precisely and would contribute to conduct culturally responsive empirical research in the field of counseling psychology. In addition, this rigorous approach can be used as a model in testing the cultural equivalence of measurements.

### Practical Implications

The theoretical concept of attachment is well known to be universal, but empirical studies demonstrate how culture-specific prototypes of attachment expressed in non-Western countries are scant. This study reviews how the prototypes of attachment can vary or be similar depending on culture by showing that different items were selected in shortening the ECR-S across countries (see Appendix Table 4). Thus, practical implications are provided for utilizing the concept of attachment in counseling practices by helping practitioners become more culturally responsive to how their clients express their attachment prototypes depending on their cultural identity. By having this culturally responsive attachment measurement, clinicians working with clients having relationship issues can help them to identify their attachment style more accurately and work on their relationship issues.

This study also considers gender differences in the expression of prototypes of attachment. In the anxiety subscale, males more easily felt that their partners changed their feelings with no apparent reason, whereas females were more likely to be upset for not obtaining the affection and support they need. These results are consistent with previous classical research that demonstrate how traditional gender norms are reflected in attachment prototypes (Ko, 1994). In this study, we tested possible gender differences by using DIF analysis and ensured that the derived scores are comparable across gender groups. When clinicians manage insecure adult attachment issues in romantic relationships, they can pay attention to how clients’ gender norms are reflected in expressing their insecure relationship with their partner and provide culturally responsive intervention. For example, for anxiously attached male clients, exploring their perceptions of their partners changing feelings with no apparent reason may be related to their inability to capture interpersonal cues. By comparison, insecurely attached female clients with romantic relationship issues are likely to be afraid of not obtaining their partners’ affection or have difficulty in disclosing their attachment related needs to their partners, which may be rooting from one’s internalized traditional gender stereotype. Thus, the social influence of traditional gender norms in forming their self-concept and in their relationship dynamic (Ahn, 2011) could be explored by using this newly developed culturally responsive measurement.

### Limitations and Future Suggestions

Despite the uniqueness of this study, several limitations require consideration. First, even if K-ECRR-SF is developed based on theoretical and empirical evidence, the interpretation and generalization of the results from this study require caution because our sample size is relatively small with participants mostly in their 20 s. Thus, further study with a larger sample or diverse age range is necessary because having a romantic partner has no age limits. Furthermore, although this study considers cultural and gender differences in item selection, the interaction between culture and gender may need further assessment in future study. Moreover, the detected gender differences in a few omitted items appear to reflect gender stereotypes and roles. Although gender identities are a spectrum and can vary, the traditional dichotomous thinking remains in Korea and is therefore explored in this study. However, to consider cultures where diverse gender identities are more accepted can also be considered. Thus, future study is needed to explore the interactions between adult attachment style, biological sex, gender roles, and sexual orientation. Finally, applications of multidimensional IRT analysis may be considered in the future to comprehensively evaluate anxiety and avoidance attachment.

## Appendix

Table 4

**Table 4:** Item selection for short versions of ECR-R in different countries

Item No. and Item		Country			
		A	B	C	D
*Anxiety*					
1	I am afraid that I will lose my partner’s love	X	X	O	X
2	I often worry that my partner will not want to stay with me	O	O	O	O
3	I often worry that my partner doesn’t really love me	O	O	X	O
4	*I worry that romantic partners won’t care about me as much as I care about them*	O	X	O	O
5	I often wish that my partner’s feelings for me were as strong as my feelings for him or her	X	O	X	O
6	I worry a lot about my relationships	O	O	X	O
7	*When my partner is out of sight, I worry that he or she may become interested in someone else*	O	X	X	O
8	*When I show my feelings for romantic partners, I’m afraid they will not feel the same about me*	O	X	X	O
9R	I rarely worry about my partner leaving me	X	X	X	X
10	*My romantic partner makes me doubt myself*	O	X	X	X
11R	*I do not often worry about being abandoned*	X	X	X	X
12	I find that my partner(s) doesn’t (don’t) want to get as close as I would like	X	O	O	X
13	Sometimes, romantic partners change their feelings about me for no apparent reason	O	X	X	X
14	*My desire to be very close sometimes scares people away*	X	O	X	X
15	I am afraid that once a romantic partner gets to know me, he or she won’t like who I really am	X	O	O	O
16	I become mad that I do not get the affection and support I need from my partner	X	O	O	X
17	I worry that I won’t measure up to other people	O	X	X	O
18	My partner only seems to notice me when I’m angry	X	X	X	X
*Avoidance*					
19	I prefer not to show a partner how I feel deep down	O	X	X	X
20R	*I feel comfortable sharing my private thoughts and feelings with my partner*	O	O	O	O
21	I find it difficult to allow myself to depend on romantic partners	X	X	X	X
22R	*I am very comfortable being close to romantic partners*	X	O	X	X
23	I don’t feel comfortable opening up to romantic partners	X	X	X	X
24	*I prefer not to be too close to romantic partners*	X	X	O	O
25	I become uncomfortable when a romantic partner wants to be very close	X	X	O	O
26R	I find it relatively easy to get close to my partner	O	O	X	O
27R	It is not difficult for me to get close to my partner	O	X	X	X
*28R*	*I usually discuss my problems and concerns with my partner*	O	O	X	O
*29R*	*It helps to turn to my romantic partner in times of need*	O	O	X	O
30R	I tell my partner just about everything	O	X	X	O
31R	I talk things over with my partner	O	O	O	O
32	I am nervous when partners get too close to me	X	X	X	X
*33R*	*I feel comfortable depending on romantic partners*	X	X	O	X
34R	I find it easy to depend on romantic partners	O	X	X	X
35R	It is easy for me to be affectionate with my partner	X	O	O	X
36R	My partner really understands me and my needs	X	O	X	O

A = Thailand (18 items), B = Czechia (16 items), C = Germany (12 items), D = Spain (18 items)

* Items in italic font indicate the ones included in K-ECRR-SF
